# Hepatitis B Antigenaemia, Nephrotic Syndrome and Pulmonary Embolism A Series of Unfortunate Events: A Case Report

**DOI:** 10.1002/rcr2.70145

**Published:** 2025-03-10

**Authors:** Muhammad Hassaan Malik, Muhammad Shaheer Bin Faheem, Khawaja Shaheryar, Javed Iqbal, Syed Aun Bin Nasar

**Affiliations:** ^1^ Department of Internal Medicine Shifa International Hospital Islamabad Pakistan; ^2^ Department of Internal Medicine Karachi Institute of Medical Sciences, KIMS Karachi Pakistan; ^3^ Department of Internal Medicine POF Hospital Wah Cantt Pakistan; ^4^ Nursing Department Communicable Disease Centre Hamad Medical Corporation Doha Qatar

**Keywords:** glomerulonephritis, hepatitis B antigenaemia, membranous nephropathy, nephrotic syndrome, pulmonary embolism

## Abstract

Hepatitis B is a major health concern. It primarily affects the liver, but it can also cause systemic complications like antigenaemia and nephrotic syndrome. Nephrotic syndrome causes proteinuria, hypoalbuminaemia, oedema, hyperlipidaemia and hypercoagulability. This hypercoagulable state may lead to a life‐threatening complication of nephrotic syndrome: thromboembolism. This case report presents a rare medical case where a patient with chronic HBV develops nephrotic syndrome and subsequent pulmonary embolism. The co‐occurrence of these diseases in a single patient highlights the importance of their intricate pathology. By examining this unique case, we aim to highlight the diagnostic and therapeutic challenges in such clinical presentations, providing valuable insight for professionals dealing with similar cases.

## Graphical abstract

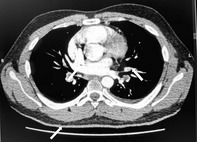

## 1 Introduction

Hepatitis B is a significant health concern, affecting millions globally [1]. While its primary effect is on the liver, HBV can also manifest as extracellular complications, such as antigenaemia, which is the presence of the hepatitis B surface antigen (HBsAg) in the serum. It indicates that the individual's liver is infected with the hepatitis B virus. The live virus continues to influence the host hepatocytes to manufacture HBsAg and can affect the kidneys, leading to nephropathy [2]. Nephrotic syndrome, one of the manifestations of HBV, is characterised by proteinuria, hypoalbuminaemia, oedema and hyperlipidaemia and is a renal disorder with diverse aetiologies. Thromboembolism, due to hypercoagulability from nephrotic syndrome [3], further adds complexity to the clinical picture. Although each condition has a distinct pathophysiology, its occurrence in a single patient presents a unique diagnostic and clinical challenge. In this case report, we present an interesting case where a patient with chronic hepatitis B infection develops antigenaemia and nephrotic syndrome and subsequent pulmonary embolism, highlighting the intricate interplay between viral hepatitis, renal disease and hypercoagulability.

## 2 Case Report

A 24‐year‐old Asian man with a previous history of hepatitis B antigenaemia presented with complaints of severe pleuritic chest pain, shortness of breath and hemoptysis. A review of the system was positive for orthopnoea, tachycardia and tachypnoea. There was a positive personal history of kidney disease.

Emergency room vitals included an elevated pulse of 122 beats per minute, blood pressure of 122/99 mmHg and respiratory rate of 27 breaths per minute. Physical examination was notable for pedal oedema. ECG revealed a q3t3 pattern.

Well's score on arrival was 5.5 for pulmonary embolism. Further lab investigations were significant for raised D‐dimer levels of 4100, proteinuria and haematuria.

**FIGURE 1:**
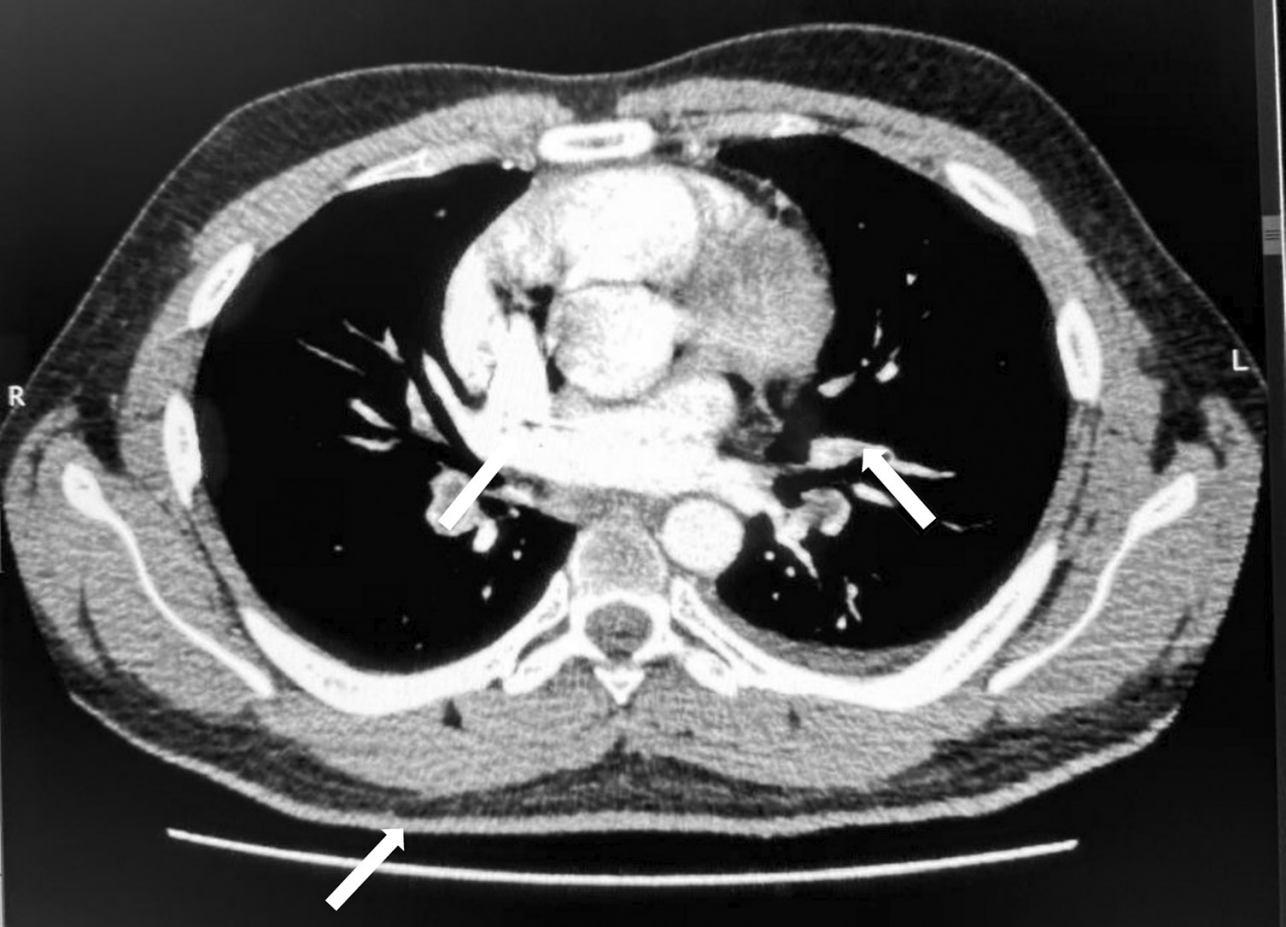
CTPA showing the presence of bilateral pulmonary embolism.

In addition, CTPA confirmed the presence of bilateral pulmonary embolism, shown in Figure 1. The patient was admitted for acute pulmonary embolism. He was started on oxygen therapy along with pain management and low molecular weight heparin. The patient gradually improved and was discharged on oral rivaroxaban. The patient was worked up for nephrotic syndrome complicated by thromboembolic events. The workup revealed a positive anti‐PLA2R antibody (43.2) with high specificity for membranous nephropathy, and 24‐h urinary proteins showed an increased level of 6820 mg/day. A renal biopsy was not performed; renal artery Doppler Ultrasound showed Grade II renal parenchymal disease with preserved CMD. The infectious workup was positive for chronic hepatitis B and reactive serum hepatitis D antibody.

The patient was started on immunosuppression (tacrolimus), Rosuvastatin, Entecavir and prednisolone. The patient tolerated the management and has since improved clinically.

## 3 Discussion

Hepatitis B antigenaemia, nephrotic syndrome and thromboembolism in a single patient present a fascinating medical case that warrants further exploration. This unique and exciting case provides insight into the pathophysiology of individual diseases and challenges traditional medical and therapeutic practices (Tables 1 and 2).

**TABLE 1:** Initial laboratory investigations.

Lab investigations	Day 1	After 4 months
Sodium	141 mmol/L	137 mmol/L
Potassium	3.8 mmol/L	4.1 mmol/L
Chloride	115 mmol/L	103 mmol/L
Blood urea nitrogen	10 mg/dL	
Urea	23 mg/dL	23 mg/dL
Serum creatinine	0.84 mg/dL	0.7 mg/dL
Calcium	2.06 mmol/L	2.5 mmol/L
Phosphorus		
Brain natriuretic peptide	< 25.0 pg/mL	
Troponin	< 0.05	
White blood cells count	13.5 × 10^3^/μL	10.9 × 10^3^/μL
Red blood cells count	5.2 × 10^6^/μL	5.4 × 10^6^/μL
Haemoglobin	15.4 g/dL	16 g/dL
Haematocrit	49%	48%
Mean corpuscular volume	95 fL	89 fL
Reticulocytes		
Platelets	97 × 10^3^/μL	160 × 10^3^/μL
HBA1C	5.3%	5.4%
D dimers	4140.1 ng/mL	93.2 ng/mL
Albumin	2.01 g/dL	4 g/dL
Anti HCV ELISA	Non‐reactive	Non‐reactive
HBSAG (ELISA)	Reactive	Reactive
HBV DNA PCR viral load	2106	
Serum T3	1.47 nmol/L	1.89 nmol/L
Serum T4	4.47 μg/dL	6.7 μg/dL
TSH	9.90 μLU/mL	1.05 μLU/mL
Anti‐thyroglobulin	7.7 IU/mL	
Anti‐thyriod peroxidase	10.10 IU/mL	
CPK	65.9 μ/L	
LDH	387 μ/L	
AST	21 U/L	
CRP	126.7 mg/L	
PT	12.5 s	
APTT	32 s	
INR	1.26	
S. total bilirubin	0.37 mg/dL	0.2 mg/dL
SGPT	12 U/L	37 U/L
Alkaline phosphatase	92 U/L	53 U/L
Serum cholesterol	234 mg/dL	140 mg/dL
Triglycerides	156 mg/dL	176 mg/dL
Uric acid	5.75 mg/dL	6.4 mg/dL
URINE D/R		
pH	5.5	
Protein	(+++)	
Glucose	Negative	
Acetone	Negative	
Blood	(+2)	
Nitrite	Negative	
Pus cells	2–4	
Red cells	Numerous	

**TABLE 2:** Proteinuria workup.

Laboratory investigations	Day 1	
	Results	
Anti‐PLA 2R antibodies	43.2 (positive)	
Urinary P/C ratio	8.52 mg/mg	
24 h urinary proteins	20,216 mg/day	495 mg/day

Although uncommon, hepatitis B antigenaemia is a problematic complication of chronic hepatitis B. HBV has been reported to form immune complexes that may deposit in the kidneys' glomeruli, resulting in various types of glomerulonephritis (GN). HBV‐GN can present with moderate to severe proteinuria, nephrotic syndrome and haematuria and can lead to membranous nephropathy [4].

Though the mechanism of thromboembolic events in nephrotic syndrome is not fully understood yet, Mirrakhimov et al. (2014) show that a loss of anticoagulants and increased synthesis of procoagulant precursors might be likely mechanisms, and this can lead to serious complications such as pulmonary embolism [5]. Membranous nephropathy, massive proteinuria and hypoalbuminemia have been identified as independent risk factors for these thromboembolic events in nephrotic syndrome. This patient had all three of these risk factors.

Leslom et al. (2020) indicate that the risk of thromboembolism in nephrotic syndrome is higher in adults (9%) compared with the paediatric population (1%), and it can lead to mortality if left unmanaged [6]. The first month after nephrotic syndrome is very critical as the patients are susceptible to most thromboembolic events within this period, but these are the highest within the first 6 months of the diagnosis.

In some patients, these thromboembolic events precede the diagnosis of nephrotic syndrome and trigger investigations leading to that diagnosis. This was the scenario in our patient, where chronic hepatitis B and nephrotic syndrome led to the development of pulmonary embolism, and further workup revealed the diagnosis of membranous nephropathy.

The presence of all these features in a single patient at such a young age prompts the need for early workup of patients with nephrotic syndrome, especially those having hepatitis B, so that thromboembolic events can be prevented [7]. Prophylactic anticoagulant therapy should be strongly considered in patients diagnosed with nephrotic syndrome to reduce the risk of thromboembolism. Severe complications like thromboembolism might be the initial presentation of a patient suffering from nephrotic syndrome without prior workup. Hepatitis B antigenaemia should prompt healthcare workers to screen patients for nephrotic syndrome and start prophylactic anticoagulation to mitigate the risk of adverse thromboembolism [8]. Similarly, retrograde workup of nephrotic syndrome in patients presenting with thromboembolism is of essence to ensure a better quality of healthcare.

## Author Contributions

**Muhammad Hassaan Malik:** conceptualization, investigation, writing – original draft, visualisation, supervision. **Muhammad Shaheer Bin Faheem:** validation, investigation, writing – original draft, writing – review and editing, supervision, project administration. **Khawaja Shaheryar:** methodology, validation, writing – original draft. **Javed Iqbal:** supervision. **Syed Aun Bin Nasar:** methodology and investigation.

## Ethics Statement

This case report was conducted in accordance with ethical principles in medical research and with the patient’s written informed consent.

## Conflicts of Interest

The authors declare no conflicts of interest.

## Data Availability

The data that support the findings of this study are available from the corresponding author upon reasonable request.
